# Intratumoral Delivery of Doxorubicin on Folate-Conjugated Graphene Oxide by In-Situ Forming Thermo-Sensitive Hydrogel for Breast Cancer Therapy

**DOI:** 10.3390/nano7110388

**Published:** 2017-11-14

**Authors:** Yi Teng Fong, Chih-Hao Chen, Jyh-Ping Chen

**Affiliations:** 1 Department of Chemical and Materials Engineering, Chang Gung University, Taoyuan 33302, Taiwan; evausatw@cgmh.org.tw; 2 Department of Plastic and Reconstructive Surgery and Craniofacial Research Center, Chang Gung Memorial Hospital, Linkou, Kwei-San, Taoyuan 33305, Taiwan; cjh5027@cgmh.org.tw; 3 Research Center for Chinese Herbal Medicine and Research Center for Food and Cosmetic Safety, College of Human Ecology, Chang Gung University of Science and Technology, Kwei-San, Taoyuan 33302, Taiwan; 4 Department of Materials Engineering, Ming Chi University of Technology, Tai-Shan, New Taipei City 24301, Taiwan

**Keywords:** thermo-sensitive hydrogel, graphene oxide, folic acid, intratumoral delivery, cancer therapy

## Abstract

By taking advantage of the pH-sensitive drug release property of graphene oxide (GO) after intracellular uptake, we prepared folic acid (FA)-conjugated GO (GOFA) for targeted delivery of the chemotherapeutic drug doxorubicin (DOX). GOFA-DOX was further encapsulated in an injectable in-situ forming thermo-sensitive hyaluronic acid-chitosan-*g*-poly(*N*-isopropylacrylamide) (HACPN) hydrogel for intratumoral delivery of DOX. As the degradation time of HACPN could be extended up to 3 weeks, intratumoral delivery of GOFA-DOX/HACPN could provide controlled and targeted delivery of DOX through slow degradation HACPN and subsequent cellular uptake of released GOFA-DOX by tumor cells through interactions of GOFA with folate receptors on the tumor cell’s surface. GOFA nano-carrier and HACPN hydrogel were first characterized for the physico-chemical properties. The drug loading experiments indicated the best preparation condition of GOFA-DOX was by reacting 0.1 mg GOFA with 2 mg DOX. GOFA-DOX showed pH-responsive drug release with ~5 times more DOX released at pH 5.5 than at pH 7.4 while only limited DOX was released from GOFA-DOX/HACPN at pH 7.4. Intracellular uptake of GOFA by endocytosis and release of DOX from GOFA-DOX in vitro could be confirmed from transmission electron microscopic and confocal laser scanning microscopic analysis with MCF-7 breast cancer cells. The targeting effect of FA was revealed when intracellular uptake of GOFA was blocked by excess FA. This resulted in enhanced in vitro cytotoxicity as revealed from the lower half maximal inhibitory concentration (IC50) value of GOFA-DOX (7.3 μg/mL) compared with that of DOX (32.5 μg/mL) and GO-DOX (10 μg/mL). The flow cytometry analysis indicated higher apoptosis rates for cells treated with GOFA-DOX (30%) compared with DOX (8%) and GO-DOX (11%). Animal studies were carried out with subcutaneously implanted MCF-7 cells in BALB/c nude mice and subject to intratumoral administration of drugs. The relative tumor volumes of control (saline) and GOFA-DOX/HACPN groups at day 21 were 2.17 and 1.79 times that at day 0 with no significant difference. In comparison, the relative tumor volumes of treatment groups at the same time were significantly different at 1.02, 0.67 and 0.48 times for DOX, GOFA-DOX and GOFA-DOX/HACPN groups, respectively. The anti-tumor efficacy was also supported by images from an in vivo imaging system (IVIS) using MCF-7 cells transfected with luciferase (MCF-7/Luc). Furthermore, tissue biopsy examination and blood analysis indicated that intratumoral delivery of DOX using GOFA-DOX/HACPN did not elicit acute toxicity. Taken together, GOFA-DOX/HACPN could be deemed as a safe and efficient intratumoral drug delivery system for breast cancer therapy.

## 1. Introduction

In recent years, an effective cancer treatment platform has always been the focus of developing advanced drug delivery systems based on different nano-sized drug carriers [1,2]. Such systems used liposomes and/or polymer nanoparticles as the drug carrier, combining triggered release of an anticancer drug under the characteristic environment of cancer cells and protect the drug until it enters the cell to increase the intracellular drug concentration [3]. Thus, the ideal drug delivery system should be not only to improve the treatment efficacy but also to decrease the systemic toxicity effects. Graphene, a novel two-dimensional (2D) honeycomb material, has been recognized as one of the most promising nanomaterials used as a filler in polymer matrices [4]. With a 2D planar structure composed of sp^2^ mixed-layer trajectories, graphene-based nanomaterials have been widely studied for applications in biotechnology [5]. When used as a drug carrier, graphene is usually modified to increase its hydrophilicity and reduce the thickness by converting it into an oxidized form (i.e., graphene oxide, GO) [6,7]. Indeed, GO was shown to be a functional nano-sized carrier for delivery of anticancer drugs based on π–π stacking, such as camptothecin (camptothecin, CPT), camptothecin derivatives (SN38) and adriamycin (doxorubicin, DOX) [8]. The hydrogen bond interactions between GO and the drug can result in a large amount of drug being adsorbed onto GO due to its large specific surface area [9,10]. An added advantage of GO for chemotherapeutic drug delivery is the pH-dependent drug release behavior, where enhanced drug release at a low pH value (pH 5.0 to pH 5.5) will provide efficient intracellular drug release after its endocytosis by the cell for drug release in the endosome [11,12]. 

Targeted delivery of anticancer drugs for cancer therapy could be more effective than traditional chemotherapy. The targeting therapy could be divided into active targeting and passive targeting [13,14]. For active targeting therapy, the drug carrier is modified with a ligand or a monoclonal antibody on the surface to increase the ability of the carrier to be specifically recognized by diseased cells. Folic acid (FA) is a group of water-soluble vitamin B that exists in green leaves, vegetables and other plants. It is an important element for all cells and involved in DNA synthesis or cell division. Folic acid is transported into healthy cells or cancer cells through their folate receptors on cell surface. As cancer cells require more FA for maintaining cell differentiation and proliferation, there are over-expressed folate receptors on the cell membrane of cancer cells, compared with healthy and/or normal cells [15]. Thus, modifying an anticancer drug nano-carrier, such as GO, with FA could enhance its ability to be recognized and its intracellular uptake efficiency by cancer cells through ligand-mediated targeting drug delivery [16,17]. Previously, we have used FA-conjugated multi-walled carbon nanotubes (a 1D carbon nanomaterial) for targeted delivery of DOX to cancer cells [18].

In-situ forming thermo-sensitive hydrogel undergoes physical sol-to-gel phase transition as temperature increases. It can be easily administered via injection using a conventional syringe needle after in-situ gelation at the physiological temperature [19]. Poly(*N*-isopropylacrylamide) (PNIPAm) is one of the most studied thermo-sensitive hydrogel showing reversible sol-gel phase transition behavior around its lower critical solution temperature (LCST) at ~32 °C [20,21]. PNIPAm end capped with a carboxylic acid group could be synthesized for subsequent conjugating with carbohydrate polymers, e.g., chitosan and hyaluronic acid, to form injectable thermo-sensitive copolymer, hyaluronic acid-chitosan-*g*-poly(*N*-isopropylacrylamide) (HACPN) with a similar LCST to PNIPAm [22]. 

Compared to traditional intravenous administration of anticancer drugs, intratumoral drug delivery systems have the potential to enable the loading and release of insoluble anticancer drugs through in-situ forming thermo-sensitive hydrogel. This drug delivery system can deliver anticancer drugs locally to the tumor site, leading to low dose requirements and reduce multiple drug administration cycles, which could reduce or eliminate adverse effects of the drug due to local delivery and prevention of systemic drug uptake [23]. Injectable gelling depots with thermo-sensitive hydrogel and pre-shaped implant systems are two types of intratumoral delivery systems for anticancer drugs [24]. The injectable gelling depot based on in-situ phase separation of thermo-sensitive hydrogel has been shown to be less invasive and lead to less pain upon injection as compared to pre-formed implants, making them a desirable system for local administration of anticancer drugs [25]. A typical injectable gelling depot system is formulated by simple mixing of drug and polymer solution below the LCST of the polymer hydrogel. After injection, sol-gel transition occurs to transform the minimally viscous solution into a drug delivery gel depot. The advantage of this method is the avoidance of invasive surgery for implantation, a high water content of the hydrogel to improve the compatibility, biodegradability of the thermo-sensitive polymer for excretion from the body once achieving its intended purpose and flexibility of the design of the drug release rate by changing the formulation [26]. 

**Figure 1:**
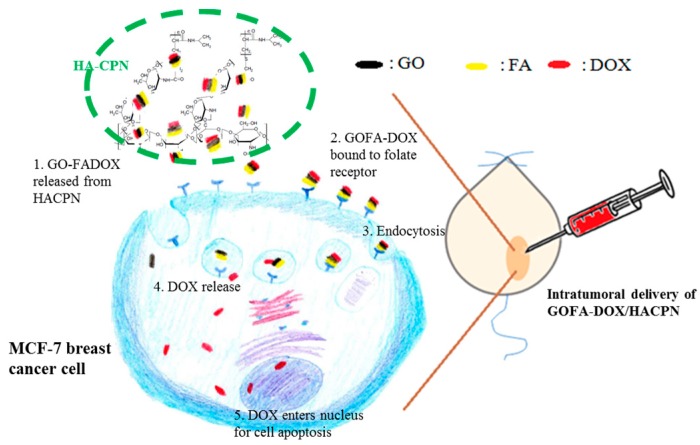
The schematic illustration of the antitumor effect by intratumoral delivery of DOX-loaded GOFA in HACPN hydrogel (GOFA-DOX/HACPN) in a xenograft tumor mouse model with MCF-7 cells implanted subcutaneously in nude mice.

However, thermo-sensitive hydrogels present challenges in anticancer drug delivery applications, i.e., initial burst release [27]. The burst release may lead to systemic toxicity due to the high dosage of drug released. The main reasons for burst release stems from the fact that a solid gel is not formed immediately upon injection into the body. A highly hydrophilic drug trapped in the aqueous phase of the gel may diffuse into the body fluid uncontrollably fast before and after gelation induced by a temperature change. To solve the burst release problem, we postulate that embedding drug-loaded GO in in-situ forming HACPN hydrogel could provide an ideal drug delivery platform for intratumoral delivery of anticancer drugs. A key requirement of in-situ depot-forming systems for local delivery, and more specifically for intratumoral delivery, could be fulfilled easily by HACPN hydrogel with its injectability through standard gauge needles [28]. Therefore, we first prepared FA-conjugated GO as the targeted drug delivery carrier for doxorubicin (DOX) (GOFA-DOX). Then, GOFA-DOX was encapsulated into the thermo-sensitive and biodegradable polymer hydrogel HACPN for local drug delivery. We demonstrated that GOFA, with its high loading capacity for DOX, showed enhanced intracellular uptake by breast cancer cell MCF-7 and pH-responsive drug release. The targeted drug delivery in concomitant with the degradation of HACPN could alleviate burst DOX release and enhance cytotoxicity toward MCF-7 cells in vitro. Furthermore, an efficient and safe breast cancer therapy employing intratumoral delivery of GOFA-DOX/HACPN in xenograft tumor mouse models with MCF-7 implanted subcutaneously in nude mice could be expected (Figure 1).

## 2. Results and Discussion

### 2.1. Synthesis and Characterization of GO and GOFA

**Figure 2:**
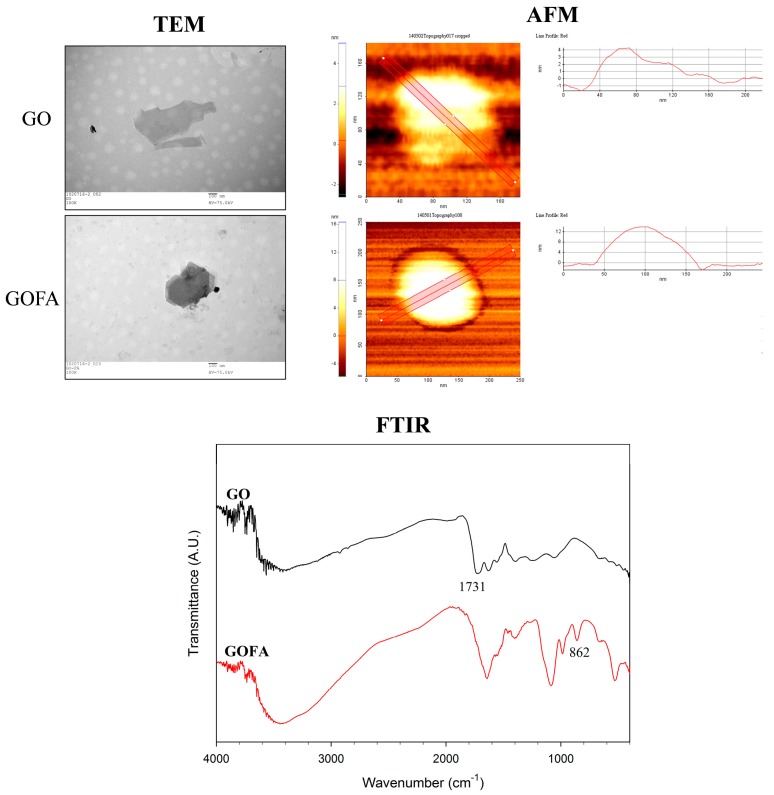
Transmission electron microscopic (TEM), atomic force microscopic (AFM) and Fourier transform infrared (FTIR) spectroscopic analysis of GO and GOFA.

The nano-sized GO was prepared by using commercial GO as raw material through modified Hummer method, followed by prolong (30 min) ultrasonication [29]. This reduced GO size and introduced abundant carboxyl groups for conjugating with FA. The morphology of as-prepared GO was characterized by transmission electron microscope (TEM) and atomic force microscope (AFM). As shown in Figure 2, the size of GO was 150~200 nm while the thickness was about 4.0 ± 0.2 nm, which was in agreement with previous studies [30]. Using 1-ethyl-3-(3-dimethylaminopropyl) carbodiimide (EDC) as a crosslinking agent, we synthesized GOFA by covalently conjugating GO with FA through amide bond formation between the amine groups of FA and carboxyl groups of GO in an aqueous solution. The EDC-mediated conjugation works by activating carboxyl groups for direct reaction with primary amines via amide bond formation. Because no portion of the EDC chemical structure becomes part of the final bond between GO and FA, it is considered a zero-length carboxyl-to-amine crosslinker. GOFA showed a light brownish color stable solution in phosphate buffered solution (PBS) with no aggregation occurred up to 1 day at 0.1 mg/mL, ensuring proper suspension during DOX loading. After FA was conjugated to GO to form GOFA, the size remained the same but the thickness increased to 12.0 ± 0.3 nm from TEM and AFM observation (Figure 2). The roughness also increased from 1.5 ± 0.4 nm to 5.3 ± 0.5 nm. Controlling the size of GOFA within 200 to 500 nm was very important for its efficient intracellular uptake into cells [31].

The modified Hummer method used here oxidized commercial GO with concentrated sulfuric acid and introduced more oxygen molecules, including abundant carboxyl groups, to GO. An aqueous suspension of GO exhibits a zeta potential of −33.0 ± 1.1 mV, indicative of negatively charged surfaces caused by the presence of hydrophilic carboxyl groups. The zeta potential of GO changed to −24.7 ± 0.9 mV after conjugation with FA as the carboxyl groups was consumed after reacting with the primary amine groups of FA. The changes in thickness, roughness and zeta potential indicated successful conjugation of FA with GO nano-sheet. 

From Fourier transform infrared (FTIR) spectroscopy analysis, major peaks of GO at 3400 cm^−1^ (OH), 1731 cm^−1^ (C=O), 1640 cm^−1^ (C=C), 1246 cm^−1^ (C–OH) and 1060 cm^−1^ (C–O) were identified (Figure 2). After FA conjugation to GO, the absorption peaks at 1731 cm^−1^ disappeared due to consumption of carboxylic acid C=O with concomitant appearance of the additional aromatic C–H bending at 862 cm^−1^ due to FA. Taken together, the FTIR analysis indicated successful incorporation of FA in GOFA. This was also supported by quantitative analysis of the amount of FA conjugated to GO, which is 98.2 μg FA/mg GOFA with 92.8% loading efficacy. 

### 2.2. Synthesis and Characterization of HACPN

**Figure 3:**
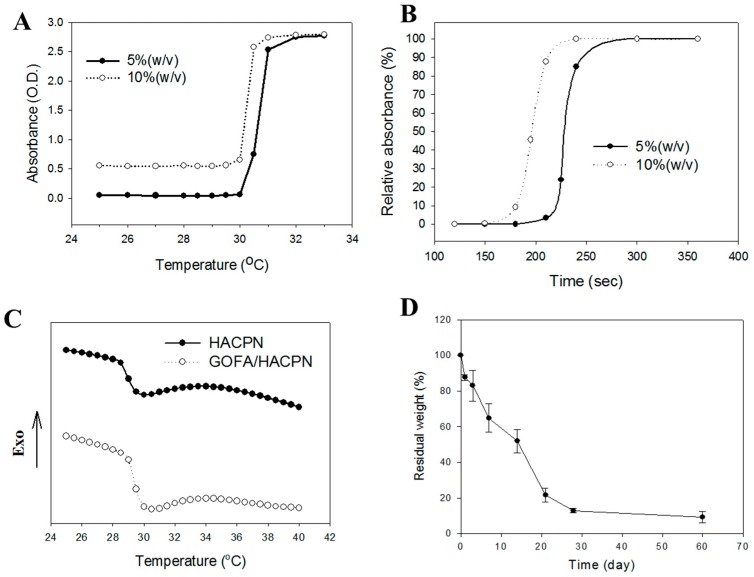
(**A**) Phase transition behavior of thermo-sensitive polymer HACPN at 5% and 10% (*w*/*v*) from 25 to 33 °C; (**B**) Kinetics of phase transition of 5% and 10% (*w*/*v*) HACPN solutions during heating with instantaneous temperature change from 25 to 37 °C; (**C**) Thermal properties of HACPN and GOFA/HACPN with 0.1% (*w*/*w*) GOFA from differential scanning calorimetry; (**D**) The weight loss of 10% (*w*/*v*) HACPN in pH 7.4 PBS at 37 °C.

Due to the toxicity of PNIPAm, copolymers containing PNIPAm and other biocompatible natural polymers were preferred for biomedical applications. Modification of PNIPAm by grafting with other biocompatible polymers could fortify the mechanical properties of the hydrogel and reduce its cytotoxicity [32]. Thus, the HACPN hydrogel is more practicable as an injectable hydrogel vehicle for drug delivery [33]. The relative compositions of chitosan, hyaluronic acid and PNIPAm in HACPN could be calculated to be 12.6% (*w*/*w*), 5.5% (*w*/*w*) and 81.9% (*w*/*w*), respectively [28]. The HACPN solution was free free-flowing at 25 °C and transformed into gels at 37 °C [32]. Furthermore, the solid hydrogel remained stationary when the sample vial was inverted, verifying the high structural strength of the injectable thermo-sensitive polymer hydrogel at the physiological temperature. The LCST was determined from the sol-gel phase transition by measuring the turbidity of a 10% (*w*/*v*) polymer solution. The relative absorbance of the polymer solution increased with temperature and the LCST could be calculated to be 30.5 and 30.3 °C for 5% and 10% (*w*/*v*) HACPN solutions, respectively, by defining the LCST being the temperature corresponding to half of the maximum change in the absorbance (Figure 3A). The gelling processes of HACPN was also thermo-reversible as subsequent cooling cycle resulted in gel-sol transition and fully reversible gel melting [28]. 

The phase transition kinetics analysis was carried out to investigate the gel formation time of HACPN solutions. As shown in Figure 3B, the relative absorbance rose sharply as the temperature was shifted from 25 to 37 °C. The gel formation kinetics of 5% HACPN was slower than that of 10% HACPN albeit both completed gel formation in less than 5 min. The fast gel formation will ensure fast in-situ gel formation to entrap DOX-loaded GOFA and prevent burst release of the drug. It should be noted that the volume of polymer solution used here (2 mL) for in vitro gel forming kinetics measurements was much larger than the volume used for in vivo injection (0.2 mL). Therefore, we expect the gel formation time will be shorter than that shown in Figure 3B (~4 min). With comparable LCST but faster gel formation kinetics, 10% HACPN was chosen for further studies. 

The effect of GOFA on the phase transition of HACPN was studied using differential scanning calorimetry (DSC). From DSC analysis (Figure 3C), the temperatures at the onsets of the differential scanning calorimetry (DSC) endotherms were at 28.70 and 29.53 °C for HACPN and GOFA/HACPN, respectively, while the corresponding peak temperatures were 29.95 and 30.07 °C, which could be referred to as the LCST [34]. Moreover, the enthalpy change of the phase transition, which was calculated by integration of peak area, increased from 0.9763 to 1.299 J/g during the heating process of the DSC cycle, indicating the replacement of water molecules around the hydrophilic polar groups by GOFA at a temperature lower than the LCST and the endothermic heat caused by the dehydration of polar groups increased [35]. 

The drug release behaviors from a hydrogel matrix depended on several factors, such as diffusion through the matrix, osmosis, degradation or weight loss of the matrix and physical parameters of the polymer matrix [36]. Taking advantage of the weight loss of HACPN hydrogel is an attractive characteristic for intratumoral drug delivery since the hydrogel does not need to be removed after local application [37]. From the weight loss at 37 °C in phosphate buffered saline (PBS), HACPN showed quick degradation rate initially with ~65% remaining weight at day 7 (Figure 3D). After this period, the degradation rate slowed down moderately with ~12% remaining weight at day 28. The degradation of HACPN hydrogel in vitro implies that GOFA/DOX could be continuously released in vivo after intratumoral delivery, followed by intracellular uptake of the nano-drug by cancer cells. 

### 2.3. DOX Loading and Release

**Figure 4:**
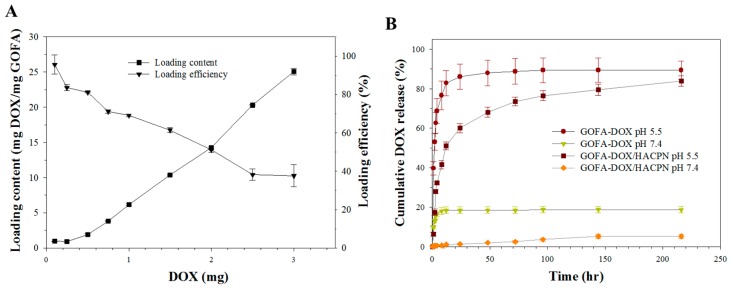
The loading and release of DOX. (**A**) Drug loading efficiency and drug loading content when 0.1 mg GOFA was reacted with different amount of DOX; (**B**) Drug release from GOFA-DOX and GOFA-DOX/HACPN at pH 7.4 and pH 5.5 in PBS (37 °C).

Drug loading and release behavior are the most important characteristics to evaluate a drug delivery system. Figure 4A shows the drug loading performance of GOFA. The high surface area and conjugate structure of GO could facilitate strong π–π stacking interactions with DOX and to achieve high DOX loadings [9]. By increasing the amount of DOX used during drug loading, the loading content (the weight of DOX to the weight of GOFA) of DOX increased sharply and reached as high as 25 mg DOX/mg GOFA when 3 mg DOX reacted with 0.1 mg GOFA. On the contrary, the DOX loading efficiency (the weight percentage of initial DOX bound to GOFA) decreased with increasing amount of DOX used and reduced from 95.5% to 37.6% when 3 mg DOX was used. Thus, reacting 2.0 mg DOX with 0.1 mg GOFA (DOX/GOFA = 20) was deemed the best condition for preparing GOFA-DOX considering both drug loading efficiency and loading content with the former being 51.2% and the latter being 14.2 mg/mg. It should be noted the DOX loading content reported here is much higher than the values (32 μg/mg and 1.84 mg/mg) reported previously using FA-conjugate carbon nanotubes [18,38]. For DOX loading to GO, the loading contents reported previously were 2.35 and 0.294 mg/mg [10,39]. These results indicated that GOFA is a highly efficient nano-carrier for loading and delivery of DOX. 

The release of drug from GOFA-DOX at 37 °C in PBS at pH 7.4 and 5.5 is presented in Figure 4B. The pH values for evaluating drug release were chosen based on the physiological and the endosomal pH value of cancer cells, respectively. The drug release curves showed that DOX loaded on GOFA was released at a slow and controlled manner at pH 7.4, to the extent of 18.7% in 216 h. The release rate of DOX was significantly enhanced at pH 5.5 and the amount of drug released was 89.4% within the same time period. 

That the release rate of DOX at pH 5.5 was significantly higher than that at pH 7.4 may be caused by weakening of hydrogen bonds between DOX and GOFA. Noncovalent attachment of DOX to GOFA involves hydrogen bonds between –COOH of GOFA and –OH of DOX and between –OH of GOFA and –OH of DOX [18]. The degree of hydrogen bond interactions between DOX and GOFA is a function of the pH value. The H^+^ in solution would compete with the hydrogen bond-forming group and weaken the hydrogen bond interactions at pH 5.5, leading to greater release of DOX. Alternatively, the high release rate at acidic conditions may be caused by the amine (–NH_2_) groups of DOX getting protonated to result in partial dissociation of hydrogel-bonding interaction [40]. The high drug loading and the pH-sensitive release of DOX suggest that GOFA is a promising delivery vehicle for the anticancer drug. 

The drug release behavior of DOX from GOFA-DOX/HACPN showed the same pH dependence as GOFA but is much slower than GOFA (Figure 4B). Thus, we can anticipate effective modulation of the burst release of DOX by entrapping GOFA-DOX in HACPN at the physiological pH extracellularly, followed by copious DOX release at the endosomal pH in cancer cells to exert enhanced cytotoxicity following the intracellular uptake of GOFA-DOX. 

### 2.4. In Vitro Cell Culture

#### 2.4.1. Cellular Uptake

**Figure 5:**
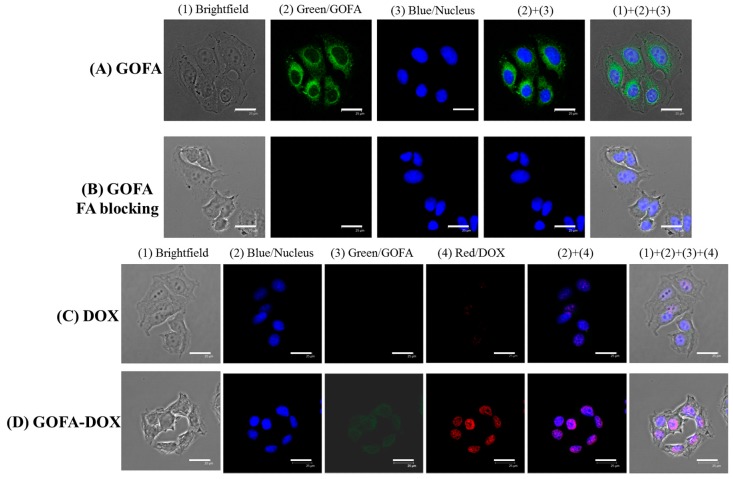
Confocal microscopy images of MCF-7 cells after incubated with GOFA for 1 h (**A**); incubated with 0.1 mg/mL folic acid for 1 h to block cell surface folate receptors and then treated with GOFA for 1 h (**B**); incubated with free DOX for 1 h (**C**); incubated with GOFA-DOX for 1 h (**D**). Bar = 25 μm.

Eukaryotic cells could form endocytotic vesicles to enclose extracellular substances through invagination of their plasma membrane segments. The specific uptake of GOFA by MCF-7 cells could be strongly suggested by receptor-mediated endocytosis to show efficient and targeted delivery of DOX by GOFA-DOX [9]. Folate receptor is a common tumor marker expressed at high levels on the surfaces of various cancer cells, which can facilitate cellular internalization of GO through receptor-mediated endocytosis after conjugating FA to GO. Confocal microscopy revealed intracellular fluorescence corresponding to quantum dot (QD)-labeled GOFA when MCF-7 cells were exposed to GOFA for 1 h and quenched with trypan blue to eliminate the residual fluorescence bound to cell membrane (Figure 5A). When folate receptors on MCF-7 cell surface were blocked by FA before contacting with GOFA, less receptor-mediated intracellular uptake was expected for GOFA. Indeed, we observed drastically diminished intracellular fluorescence signal of GOFA by blocking with excess FA (Figure 5B). This difference is due to the efficient blockage of folate receptor on MCF-7 cell surface with free FA in solution, which competitively inhibited the affinity of folate receptor toward GOFA. Overall, our result was in agreement with previous studies that revealed folate receptor-dependent cellular uptakes by cancer cells for anticancer drug delivery [41].

Two possible mechanisms of cellular DOX uptake from nanoparticles have been suggested: (1) DOX is released from the nanoparticle outside the cells, or (2) DOX is carried by the nanoparticle and released inside the cells [42]. Folic acid modification could increase the cytotoxicity of DOX encapsulated in nanoparticles toward MCF-7 cells by minimizing extracellular DOX release [43]. Upon incubation of MCF-7 with free DOX at 37 C for 1 h, red fluorescence was found mostly confined within the nucleus of shrunken cells, where DOX is chelated with DNA (Figure 5C). For GOFA-DOX, green fluorescence of GOFA appears in the cytoplasm of shrunken cells (Figure 5D). This provides a direct evidence for endocytosis of GOFA, which accumulated in the cytoplasm after internalization. Red fluorescence was only observed in the cell nucleus, indicating DOX released from GOFA in the cytoplasm could translocate across the nuclear membrane to interact with DNA molecules in the cell nucleus (Figure 5D). That the red signal due to DOX is much stronger for GOFA-DOX than DOX also implied GOFA could facilitate DOX diffusion across the cell membrane through intracellular uptake of GOFA-DOX to enhance the cytotoxicity toward cancer cells. Taken together, the results suggest that DOX-loaded GOFA could be transported across cell membrane via endocytosis and DOX was subsequently released under the acidic intracellular environment. 

**Figure 6:**
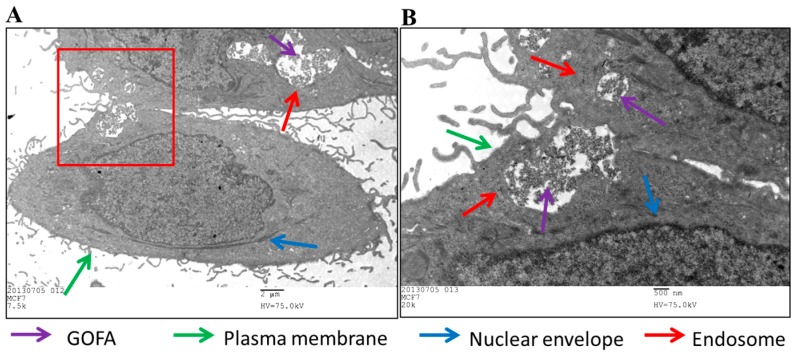
(**A**) Transmission electron microscope (TEM) micrographs of MCF-7 cells treated with GOFA for 1 h. (**B**) is an enlarged view of the square in (**A**). (**A**) Bar = 2 μm, (**B**) Bar = 500 nm.

Internalization of GOFA by MCF-7 cells was also observed by transmission electron microscope (TEM) to confirm endocytosis. Intracellular uptake was evident after contacting GOFA with cells, which were found within the endosomes in the cytoplasmic region (Figure 6). The presence of GOFA in close proximity to the nuclear region could be also observed. In vitro confocal and TEM images therefore strongly support efficient entry of GOFA into cancer cells through endocytosis after releasing from HACPN. It could be postulated that GOFA could potentially enhance the apoptotic effects of DOX via its efficient endocytosis by the cancer cells and increase the intracellular anticancer activity of the drug [44].

#### 2.4.2. In Vitro Cytotoxicity and Biocompatibility Studies

**Figure 7:**
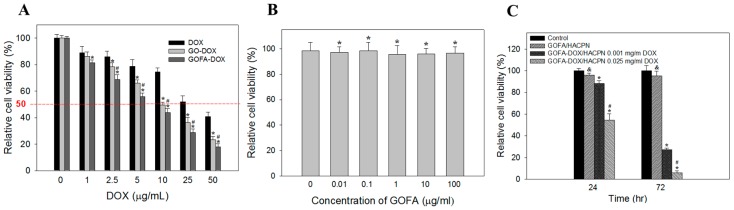
(**A**) Cytotoxicity of free DOX, GO-DOX and GOFA-DOX against MCF-7 cells. The cells were treated for 24 h. The relative cell viability was compared to the control without DOX. * *p* < 0.05 compared with DOX, ^#^ *p* < 0.05 compared with GO-DOX; (**B**) Viability of MCF-7 cells after incubated with different concentration of GOFA for 24 h. The relative cell viability was compared to the control without GOFA. * *p* > 0.05 compared with control; (**C**) Cell viability of MCF-7 cells after incubated with GOFA/HACPN and GOFA-DOX/HACPN at different DOX concentrations for 24 and 72 h. The control is cell culture medium. ^&^ *p* > 0.05 compared with control, * *p* < 0.05 compared with control, ^#^ *p* < 0.05 compared with GOFA-DOX/HACPN 0.001 mg/mL DOX. Data are presented as mean ± standard deviation (SD), *n* = 6.

After confirming the successful entry of GOFA-DOX into the cells and release the drug, cell viability assay was used to compare the cytotoxicity of GO-DOX, GOFA-DOX and free DOX at different DOX concentrations toward MCF-7 cells (Figure 7A). When treated with an equivalent concentration of DOX, MCF-7 cells showed the lowest viability when treated with GOFA-DOX, followed by GO-DOX and free DOX. The IC50 values were calculated to be 7.3, 10 and 32.5 μg/mL for GOFA-DOX, GO-DOX and free DOX, respectively. This suggests GOFA-DOX can efficiently deliver the drug to the cell nucleus area due to the high cellular internalization of FA-conjugated GO via receptor-binding endocytosis. The biocompatibility of GOFA was confirmed over a broad concentration range using MCF-7 cells (Figure 7B), where the relative cell viability is above 90% up to 100 μg/mL, which covers the concentrations of GOFA studied in Figure 7A, indicating cell cytotoxicity shown by GOFA-DOX was indeed from DOX released but not from the drug carrier itself. 

For the cytotoxicity of GOFA-DOX/HACPN toward MCF-7 cells, when cells were incubated at a low DOX concentration (0.001 mg/mL), cell survival rate was 90% after 24 h (Figure 7C). When MCF-7 cells were cultured for 72 h in the presence of GOFA-DOX/HACPN, cell viability was further decreased to ~30%. At a higher DOX concentration (i.e., 0.025 mg/mL), the same cytotoxicity effect could be observed. That the cytotoxicity of GOFA-DOX/HACPN was both DOX dose and time-dependent, indicating GOFA-DOX could be released from HACPN continuously to exert the cytotoxicity effect toward MCF-7 cells. The biocompatibility of GOFA/HACPN could be also observed from Figure 7C with the relative cell viability not significantly different from the control, indicating GOFA/HACPN does not elicit cytotoxicity toward MCF-7 cells in vitro. 

#### 2.4.3. Cell Apoptosis Induced by DOX In Vitro

**Figure 8:**
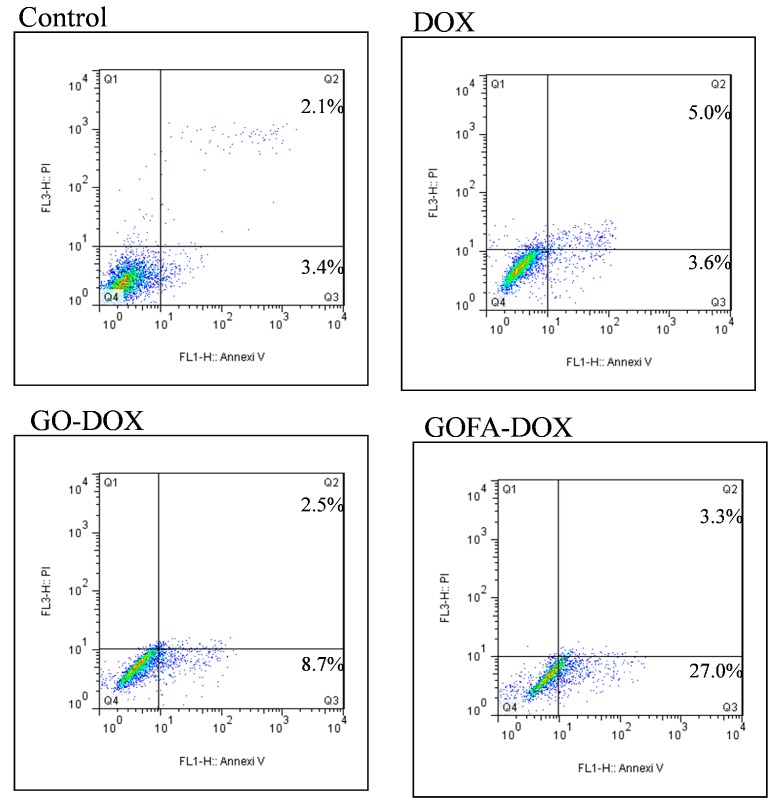
Flow cytometer analysis of the apoptotic and necrotic cells by Annexin V-FITC/PI staining (Q1: necrotic; Q2: late apoptotic; Q3: early apoptotic; Q4: live) after 24 h incubation with free DOX, GO-DOX and GOFA-DOX, respectively. The numbers in Q1 to Q3 indicate the percentage of cells after the treatment.

Doxorubicin is known as an anthracycline antibiotic effective in treating a variety of cancers. It functions primarily at the DNA level by blocking the replication and transcription processes [45,46]. DOX also activates damage-inducible DNA repair and prevent the triggering of programmed cell death by spontaneous and induced DNA damage [47]. To confirm the cytotoxicity of GO-DOX and GOFA-DOX toward MCF-7 cells was induced by apoptosis as of free DOX and compare the apoptosis ratio, Annexin V-FITC/PI staining assays was performed and the apoptotic and necrotic cells were quantified by flow cytometry. The percentages of necrotic (Q1), late apoptotic (Q2), early apoptotic (Q3) and live cells (Q4) are shown in Figure 8. The flow cytometry analysis revealed that early and late apoptosis represented the major death mode of MCF-7 cells, which was caused by free DOX or DOX released form GO (GOFA). The ratio of apoptosis cells treated with GOFA-DOX was 30.3%, compared with that of free DOX (8.6%). Most importantly, it is worth noting that the ratio of apoptosis cells treated with GOFA-DOX was markedly higher than that in cells treated with GO-DOX (11.2%), endorsing the targeting effect of FA. In general, the results of flow cytometry were consistent with the cell viability results by 3-(4,5-dimethyl-2-thiazolyl)-2,5-diphenyl-2H-tetrazolium bromide (MTT) assays (Figure 7A), underlining the importance of using GOFA to facilitate the entrance of DOX-loaded nano-carrier into the cells through endocytosis and subsequently release DOX that enters the nucleus to exert the cell cytotoxicity (Figure 5C,D). Furthermore, the FA-mediated intracellular uptake of GOFA could enhance the endocytosis of the nano-carrier to substantially increase the extent of cytotoxicity of DOX toward MCF-7 though cell apoptosis (Figure 5A,B).

### 2.5. Animal Study

#### 2.5.1. Antitumor Effect

With an aim to improve antitumor therapeutic effects and to decrease the side effects of DOX, we have successfully demonstrated that GOFA nano-carrier could conjugate with DOX and be encapsulated in HACPN to enhance its cytotoxicity toward MCF-7 breast cells in vitro. To validate those data in vivo, we administered the DOX-loaded GOFA in HACPN by taking advantage of the in-situ gelling property of the thermo-sensitive hydrogel. All BALB/c nude mice with an aggressive subcutaneous MCF-7 cells tumor were injected intratumorally with formulations containing saline (control), GOFA/HACPN, free DOX, GOFA-DOX or GOFA-DOX/HACPN, followed by measuring the tumor size and mouse body weight. In order to successful determining the anti-tumor effects in vivo, the size of the tumor was controlled within 60–100 mm^3^ when the treatment started. 

**Figure 9:**
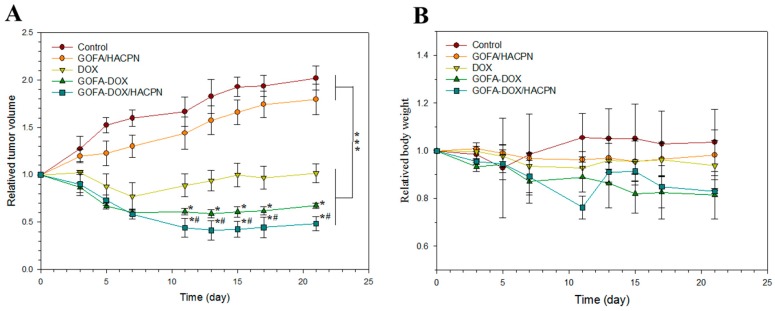
Antitumor activity induced by DOX in nude mice bearing MCF-7 cancer cells. DOX (30 mg/kg) was administered intratumorally and tumor volume (**A**) and body weight (**B**) changes were recorded. The data are shown as mean ± standard deviation (SD), *n* = 6. * *p* < 0.05 compared with DOX, ^#^ *p* < 0.05 compared with GOFA-DOX, *** *p* < 0.001.

As shown in Figure 9A, the same trends of tumor growth were observed in the control group and the GOFA/HACPN group. The relative tumor volume increased rapidly during the treatment period and reached 2.17 ± 0.02 (control) and 1.79 ± 0.16 (GOFA/HACPN) at day 21 with no significant difference between groups. In contrast, the tumor growth rate was inhibited at different levels in all DOX-treated groups. The DOX, GOFA-DOX and GOFA-DOX/HACPN groups showed 0.82 ± 0.10, 0.67 ± 0.02 and 0.48 ± 0.07 relative tumor sizes at day 21, respectively, with significant difference among groups. Indeed, in vitro cytotoxicity results also endorsed the substantial enhancement of cytotoxicity of GOFA-DOX toward MCF-7 cells over free DOX at the same drug dosage (Figure 7A and Figure 8). Intratumoral injection of DOX showed associated cytotoxic effects only at the early stage of treatment and short-term inhibition of tumor growth. The tumor volume rapidly dropped as early as 3 days after treatment and lasted for 4 days, followed by a rebound phenomenon in tumor volume at the later stage of treatment. For the GOFA-DOX group, with the targeting effect of FA, DOX could be more efficiently delivered to MCF-7 cells and the relative tumor volume could be significantly reduced throughout the test period after day 7 and showed minimal recovery 7 days after treatment. Nonetheless, the most efficient cytotoxic effect and continuous inhibition of tumor growth was observed only in the GOFA-DOX/HACPN group where the tumor size was continuously reduced up to 11 days to resulted in the highest tumor inhibition ratio of 52% (based on tumor volume changes) after 21 days, suggesting the in vivo anti-tumor efficacy using a combinatory GOFA-DOX/HACPN intratumoral drug delivery platform. These results demonstrated that thermo-sensitive HACPN hydrogel loading GOFA-DOX for cancer in-situ treatment could lead to more extensive destruction of tumor tissues and enhance the therapeutic efficiency. The enhanced intracellular uptake of GOFA-DOX contributed to the higher tumor-killing ability when compared with the free DOX dosage form. For comparison with GOFA-DOX, in-situ forming HACPN thermo-sensitive hydrogel can be retained around the tumor tissue and slowly released GOFA-DOX in concomitant with hydrogel degradation, which could raise local DOX concentration in the tumor and enhance the topical bioavailability of DOX for the best antitumor effect toward MCF-7 cells.

To assess the potential for adverse effects associated with treatments, mice were observed for changes in their body weight and appetite, for diarrhea and abnormal behavior over the course of treatments. Neither control nor drug-treated mice showed abnormalities in appetite and behavior throughout the 21 days observation period and there was no significant difference in weight for all treatment groups from the control (Figure 9B).

#### 2.5.2. Histological and Systemic Toxicity Analysis

**Figure 10:**
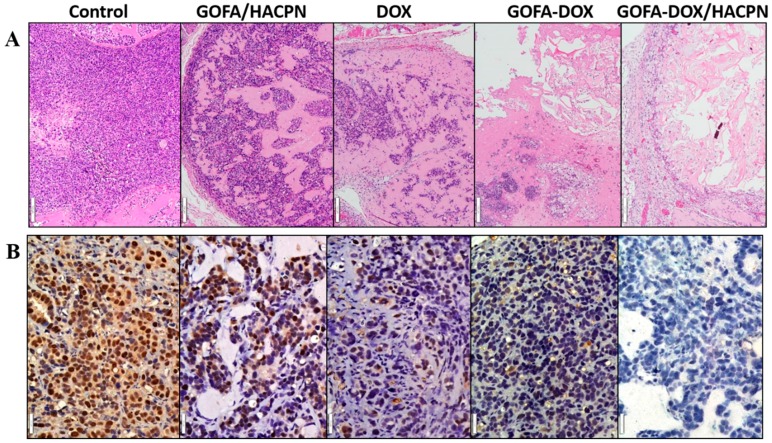
(**A**) H&E (Bar = 100 μm) and (**B**) proliferating cell nuclear antigen (PCNA) immunohistochemical (Bar = 20 μm) staining of retrieved tumor tissues at day 21.

At day 21, the tumors were harvested for histological analysis. As shown in Figure 10A, there was no evidence of necrosis in the H&E staining slide for the tumor in the control group. Minimum necrosis was observed in the GOFA/HACPN group whereas some necrosis regions were observed in the free DOX and GOFA-DOX groups. There was significantly more necrosis regions in tumors treated with GOFA-DOX/HACPN when compared with other DOX-treated groups. Indeed, the H&E staining revealed that the cavitation phenomenon in coagulative necrosis was more obvious in the GOFA-DOX/HACPN group (Figure 10A). These results demonstrated that intratumoral delivery of GOFA-DOX/HACPN enhanced the anti-tumor efficacy, suggesting it is an excellent treatment for breast cancer. 

Improved tumor delivery of DOX should also inhibit proliferation of cancer cells. Immunohistochemical examination of tumor sections associated with the cell proliferation marker- proliferating cell nuclear antigen (PCNA) clearly indicated that a greater number of actively proliferating tumor cells existed in tumor sections from the control and the GOFA/HACPN groups. In contrast, the tumor treated with DOX and GOFA-DOX showed weak PCNA immunoreactivity. The PCNA expression was the lowest in the GOFA-DOX/HACPN group than other groups (Figure 10B). Therefore, we conclude GOFA-DOX/HACPN can provide effective anticancer activity to effectively inhibit proliferation of cancer cells [48]. 

**Figure 11:**
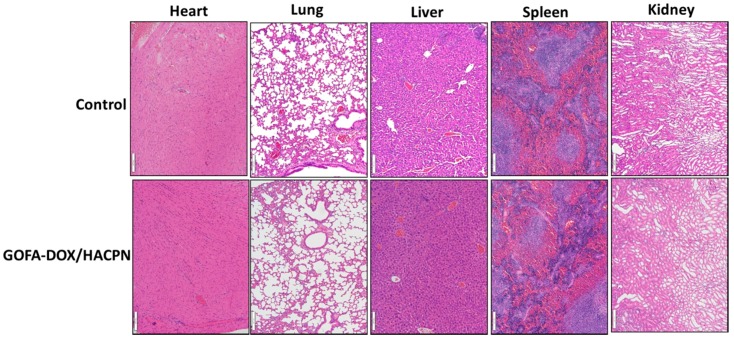
Histological examination of heart, lung, liver, spleen and kidney tissues by H&E stain after euthanization at day 21. Tissue biopsy did not reveal any observable differences between the control and the GOFA-DOX/HACPN group. Bar = 100 μm.

When the animals were euthanized, no gross abnormalities were observed in the treated mice. To further assess possible systemic toxicity, the major organs for the mice treated with GOFA-DOX/HACPN at day 21 were harvested for morphologic evaluation of H&E-stained sections and compared with those in the control group. As shown in Figure 11, the GOFA-DOX/HACPN group did not reveal any observable differences from the control group based on the histological examination of heart, lung, liver, spleen and kidney biopsy. H&E staining of the heart tissue sections showed striated cardiac muscles with the centrally placed nucleus. Normal alveoli without the sign of pulmonary fibrosis were seen in the lung sections. The histology of liver tissues revealed normal hepatocytes, central veins, portal triads and liver lobules. Red pulp and white pulp appeared in spleen samples. The kidney biopsy samples contained normal Bowman’s capsule surrounding glomeruli as well as convoluted tubules. That the GOFA-DOX incorporated HACPN hydrogels exhibit reduced systemic toxicities could be due to the localized and delayed release of DOX encapsulated in HACPN at the tumor site for good biocompatibility and safety.

On blood sampling of animals after the experiment, the application of GOFA-DOX/HACPN did not significantly alter the level of blood counts and hepatic or renal functions from hematologic study when compared with the control group (Table 1). These results were consistent with the overall health of mice from histological analysis.

Clinical applications of anticancer drugs are limited by side effects such as cardiac toxicity [49]. From the safety evaluation data in Figure 11 and Table 1, the intratumoral delivery of GOFA-DOX/HACPN appeared to be well tolerated by the animals. This could be suggested to be stemmed from combined effects of intratumoral injection and in-situ forming drug delivery system. The intratumoral delivery of DOX using GOFA-DOX/HACPN could provide a high local concentration of the antitumor drug and the in-situ forming HACPN hydrogel together with GOFA-DOX would eliminate the initial burst release of DOX to reduce systemic toxicity [50]. 

Considering the toxicity of GO, it is generally considered to be safe for in vivo applications [51]. For HACPN, the biodegradable components chitosan and HA are safe science chitosan is biodegradable predominantly by lysozyme and by bacterial enzymes in the colon in vertebrates [52] while HA could be degraded through step-wise enzymatic or non-enzymatic reactions in vivo [53]. For the non-biodegradable PNIPAm component in HACPN, it was reported that low molecular weight PNIPAm showed good biocompatibility in vivo by undergoing renal clearance [54]. By using PNIPAm polymers with a low molecular weight (22 kDa) for HACPN synthesis [28], we did not expect the PNIPAm generated by HACPN degradation to exert any in vivo toxicity as it is below the renal cutoff [55].

#### 2.5.3. IVIS for Bioluminescence Imaging (BLI) Intensity

**Figure 12:**
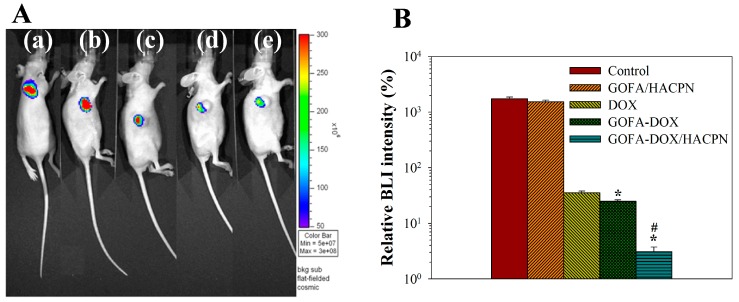
The bioluminescence imaging (BLI) of subcutaneously implanted MCF-7/Luc cells in nude mice. The BLI signal was measured at baseline (before treatment) and at day 21 after treatment. (**A**) Representative BLI obtained in the (**a**) control (saline), (**b**) GOFA/HACPN, (**c**) DOX, (**d**) GOFA-DOX and (**e**) GOFA-DOX/HACPN group by IVIS at day 21. (**B**) Plot of the relative BLI signal intensity at day 21 (mean ± SD, *n* = 6). The relative BLI signal intensity (%) was calculated from the total bioluminescent signal intensity at day 21 normalized by the total bioluminescent signal intensity at baseline. * *p* < 0.05 compared with DOX, ^#^ *p* < 0.01 compared with GOFA-DOX.

As residual hydrogel may influence the measurement of tumor volume, we further used a stably luciferase report gene-transfected MCF-7 cells (MCF-7/Luc) and in vivo imaging system (Xenogen IVIS-200, Caliper Life Sciences, Hopkinton, MA, USA) to determine the bioluminescence imaging (BLI) intensity of tumors formed from subcutaneous implanted MCF-7/Luc cells. It has been demonstrated that luciferase expression and bioluminescence does not affect tumor cell growth for MCF-7 cells [56]. For therapeutic effects from BLI imaging, the mean value of the normalized BLI signal intensity increased to 1738% in the control group after 21 days, reflecting an active tumor growth for MCF-7/Luc cells (Figure 12). Without any drug, the GOFA/HACPN treatment did not showed a significant difference in BLI signal from the control group, albeit with a lower mean BLI signal intensity of 1539%. In the DOX-treated group, the mean values decreased to 35.3% 21 days after single DOX administration, indicating that the cytotoxic effect of DOX affected tumor growth (Figure 12). In the GOFA-DOX group, the mean value further reduced to 25.0% with significant difference in BLI intensity from the DOX-treated group. However, a remarkable drop in normalized BLI signal intensity to 3.1% was observed for the combinatory GOFA-DOX/HACPN group and the BLI signal was significantly different from all DOX-treated groups. In general, the antitumor effect from different treatments with IVIS imaging was consistent with that from tumor volume change shown in Figure 9A. 

## 3. Materials and Methods

### 3.1. Materials

Graphene oxide (GO) (N002-PS) and quantum dots (QD) (QSA-490, CdSSe/ZnS core/shell QDs with amine group) were purchased from Angstron Materials (Dayton, OH, USA) and Ocean NanoTech (San Diego, CA, USA), respectively. *N*-isopropylacrylamide (NIPAM), azobisisobutyronitrile (AIBN), mercaptoacetic acid (MAA), chitosan (deacetylation degree = 98%, molecular weight = 1.5 × 10^5^ Da), 2-morpholinoethane sulfonic acid (MES), 3-(4,5-dimethyl-2-thiazolyl)-2,5-diphenyl-2*H*-tetrazolium bromide, (MTT), 2,4,6-trinitrobenzene sulfonic acid (TNBS) and 4′,6-diamidino-2-phenylindole dihydrochloride (DAPI), folic acid (FA), doxorubicin (DOX) hydrochloride were purchased from Sigma-Aldrich (St. Louis, MO, USA). 1-ethyl-3-(3-dimethylaminopropyl) carbodiimide (EDC) and *N*-hydroxysuccinimide (NHS) were obtained from Acros (Geel, Belgium). Potassium salt of D-luciferin was obtained from Gold Biotechnology, Inc. (New Taipei City, Taiwan). Hyaluronic acid (HA, sodium hyaluronate) from *Streptococcus zooepidemicus* with an average molecular weight of 1.8 × 10^6^ Da was purchased from Bloomage Freda Biopharm Co. (Jinan, China). Minimum Essential Medium (α-MEM, ThermoFisher Scientific, Waltham, MA, USA) and fetal bovine serum (FBS, HyClone, Logan, UT, USA) were used for cell culture.

### 3.2. Preparation and Characterization of GO and GOFA

#### 3.2.1. Preparation of GO, GOFA and Quantum Dot (QD)-Labeled GO and GOFA

The preparation and modification of GO followed the modified Hummers’ method [4,57]. Briefly, 1 g of GO was stirred in 23 mL sulfuric acid for 12 h, followed by slowly adding 3 g KMnO_4_ below 20 °C. The temperature was increased to 40 °C while stirring for another 30 min. The temperature was increased to 80 °C and stirred for another 45 min. 46 mL of distilled deionized water (ddH_2_O) was added and the solution was heated to 98–105 °C for 30 min, followed by cooling down to room temperature for 1 h. Additional ddH_2_O (140 mL) and 10 mL of 30% H_2_O_2_ were added and reacted for 5 min at 40 °C. After the reaction, GO was washed three times with 5% hydrochloride acid by centrifugation and dialyzed against ddH_2_O till the pH become neutral. Nano-sized GO was obtained by sonicating for 30 min at 800 W and filtered with a 0.2 μm filter and adjusted the final concentration of the GO solution to 0.2 mg/mL for future modification.

Folic acid (FA) molecules were conjugated to GO through carbodiimide-mediated covalent bonds formation between carboxyl groups in GO and amine groups in FA [9,58]. In short, 0.1 mg GO was mixed with 6 mM EDC and 6 mM NHS in 10 mL pH 6 phosphate buffered saline (PBS) for 1.5 h to activate the carboxyl groups in GO. One milliliter of FA solution (0.1 mg/mL) in pH 7.4 PBS was added to above solution and allowed to react at room temperature for 2 h to react the amine groups in FA with activated carboxyl groups in GO. After centrifugation at 14,000× *g* for 30 min, the product was washed several times with ddH_2_O to remove unreacted reagents and then dried at room temperature. The amount of FA immobilized to GO was determined by subtracting the amount of FA left in the reacting and washing solutions from the amount of FA initially added. The concentration of FA in the solution was determined by UV-Vis spectroscopy at 358 nm. 

For preparing QD-labeled GO or GOFA, 0.5 mL of GO or GOFA (1 mg/mL) was reacted with 20 μL of 8 μM QDs and 0.05 mL of 60 mM EDC and 0.05 mL of 60 mM NHS for 90 min. After centrifugation at 14,000× *g* for 30 min, the product was washed several times with PBS and dispersed in PBS for use. 

#### 3.2.2. Characterization of GO, GOFA and GOFA-DOX

An atomic force microscope (AFM) (XE-70, Park Systems, Santa Clara, CA, USA) was used to analyze the surface topography, size and thickness of samples. Diluted GO or GOFA in alcohol were deposited onto a freshly cleaved Mica substrate and imaged after alcohol evaporation. Transmission electron microscopy (TEM) images were taken using JEM-2000EXII TEM (JEOL, Tokyo, Japan). The Fourier transform infrared (FTIR) spectra were recorded on a FT-730 FTIR spectrometer (Horiba, Japan) by mixing samples with KBr and scanned from 400 to 4000 cm^−1^ at 2.5 mm/s. 

### 3.3. Preparation and Characterization of HACPN Hydrogel

#### 3.3.1. Synthesis of HACPN Hydrogel

The HACPN temperature-sensitive hydrogel was synthesized as described previously [28,59]. Briefly, NIPAM and AIBN were purified by recrystallization in n-hexane and methanol, respectively. PNIPAM end-capped with a carboxyl group (PNIPAM-COOH) was synthesized in benzene by free radical polymerization of NIPAM and MAA (chain transfer agent) in the presence of AIBN (initiator). PNIPAM-COOH was reacted with chitosan in 0.1 M MES buffer (pH 5.0) containing NHS and EDC to get chitosan-*g*-PNIPAM (CPN) copolymer. By thermally induced precipitation, the CPN copolymer was recovered by centrifugation. For HACPN synthesis, CPN copolymer was further reacted with HA in 0.1 M MES buffer (pH 5.0) containing EDC and NHS to get HACPN. Residual HA was removed by thermal precipitation of HACPN at 50 °C, followed by dialysis (molecular weight cut-off (MWCO) 300,000) at 4 °C and lyophilization. 

#### 3.3.2. Characterization of HACPN Hydrogel

To determine the LCST, 10% (*w*/*v*) polymer solutions (5% or 10% (*w*/*v*)) were prepared in ddH_2_O. The sol-gel phase transition of the polymer solution was measured using an UV-Vis spectrophotometer (Spectronic 200, Thermo Scientific, Waltham, MA, USA) equipped with a circulator bath for temperature control. A semi-micro cuvette (10 mm light path) containing 2 mL of polymer solution was used. The absorbance of the polymer solution at 470 nm was recorded from 25 to 33 °C. The polymer solution was equilibrated at each test temperature for 60 min. From the thermo-precipitation curve by plotting the absorbance at 470 nm (OD_470_) vs. temperature, the LCST of the polymer was defined as the temperature when the absorbance was half of the maximum value. 

For sol-gel phase transition kinetics, 2 mL of 5% or 10% (*w*/*v*) of polymer solutions in ddH_2_O were put in a semi-micro cuvette and sealed with Parafilm. The samples were equilibrated in a 25 °C incubator for 60 min and then placed in an UV-Vis spectrophotometer pre-equilibrated at 37 °C. The turbidity of polymer solution was recorded as a function of time up to 6 min.

For differential scanning calorimetry (DSC) analysis, 10 mg polymer solutions prepared in ddH_2_O were placed in a DSC aluminum pan and analyzed with a Q20 DSC (TA Instruments, New Castle, DE, USA). The scan rate was 1 °C/min from 10 to 40 °C under 30 mL/min nitrogen.

For degradation of HACPN, 0.2 mL of 10% (*w*/*v*) HACPN hydrogel (20 mg HACPN) was placed in a pre-weighed Millicell^®^ cell culture insert (Millipore) and immediately gelled at 37 °C. Five milliliters of PBS (pH 7.4, 37 °C) was added to the HACPN gel and the cell insert was shaken at 50 rpm in a 37 °C incubator. At different time points, samples were removed and rapidly frozen at −80 °C, followed by freeze-drying and weighing to obtain the residual weight of HACPN. The in vitro degradation of HACPN was calculated from the following equation,

(1) $$\mathrm{Residual}\;\mathrm{weight}(\%)=(\frac{W_{t}}{W_{i}})\times 100\%$$

where *W*_i_ is the initial weight of HACPN and *W_t_* is the weight of HACPN at time *t*. 

### 3.4. DOX Loading and Release

#### 3.4.1. Loading of DOX on GOFA

A solution containing DOX (0.1–3.0 mg) and 0.1 mg of GOFA was prepared in 1 mL PBS (pH 7.4) and stirred at 4 °C for 24 h in dark. The GOFA-DOX were collected by ultracentrifugation (14,000× *g* for 15 min) and washed three times with PBS until the supernatant became color-free. The amount of unbound DOX in the solution was determined by measuring the absorbance at 490 nm (OD_490_). The drug loading efficiency (%) and the drug loading content is defined as,

(2) $$\mathrm{Loading}\;\mathrm{effcienct}\;(\%)=\frac{\mathrm{Weight}\;\mathrm{of}\;\mathrm{loaded}\;\mathrm{DOX}\;(\mathrm{mg})}{\mathrm{Weight}\;\mathrm{of}\;\mathrm{initial}\;\mathrm{DOX}\;(\mathrm{mg})}\times 100\%$$

(3) $$\mathrm{Loading}\;\mathrm{content}=\frac{\mathrm{Weight}\;\mathrm{of}\;\mathrm{loaded}\;\mathrm{DOX}\;(\mathrm{mg})}{\mathrm{Weight}\;\mathrm{of}\;\mathrm{GOFA}\;(\mathrm{mg})}$$

#### 3.4.2. In Vitro DOX Release from GOFA-DOX and GOFA-DOX/HACPN

For drug release, 0.1 mg/mL GOFA-DOX was prepared in 1 mL of phosphate buffered saline (PBS) at pH 5.7 (endosomal pH) or at pH 7.4 (physiological pH) at 1.8 mg/mL DOX. The solution was shaken at 50 rpm and 37 °C, followed by ultracentrifugation to separate GOFA-DOX at pre-determined times [60]. All supernatant was removed and replenished with an equal volume of PBS of the same pH for further drug release studies. The concentration of DOX in the supernatant was quantified using an enzyme-linked immunosorbent assay (ELISA) reader at 490 nm. The DOX release results were calculated in a cumulative manner by the following equation [43],

(4) $$\mathrm{Cumulative}\;\mathrm{Dox}\;\mathrm{released}\;(\%)=(\frac{\mathrm{Cumulative}\;\mathrm{amout}\;\mathrm{of}\;\mathrm{DOX}\;\mathrm{released}}{\mathrm{Initial}\;\mathrm{amount}\;\mathrm{of}\;\mathrm{DOX}})\times 100\%$$

The DOX release from GOFA-DOX/HACPN hydrogel was determined for 10% (*w*/*v*) HACPN by dissolving 0.1 g HACPN in 1 mL GOFA-DOX solution (0.1 mg/mL in pH 7.4 PBS). 0.5 mL of HACPN solution was placed in Millicell^®^ cell culture inserts fitted in a 6-well cell culture plate. 5 mL of PBS buffer (pH = 7.4) was added to each well to totally immerse the copolymer hydrogel in the insert and the plate was incubated at 37 °C by shaking at 50 rpm. At predetermined times, all solution in each well was removed for determination of the DOX concentration using an ELISA reader at 490 nm and an equal volume of PBS buffer (pH 7.4) was added to calculate the cumulative DOX release by Equation (4).

### 3.5. In Vitro Cell Culture

#### 3.5.1. Cell Line and Cell Culture Condition

The MCF-7 human breast adenocarcinoma cell line (BCRC 60436) was obtained from the Bioresource Collection and Research Center (Hsinchu, Taiwan). The cells were cultured in α-MEM medium supplemented with 10% FBS at 37 °C in a humidified CO_2_ incubator containing 5% CO_2_. MCF-7 cells were sub-cultured routinely by using trypsin-ethylenediaminetetraacetic acid (EDTA) (Gibco, Thermo Fisher Scientific, Waltham, MA, USA) when cells reached 80–90% confluence. 

The MCF-7/Luc cell line that could stably expresses firefly luciferase and neomycin-resistant genes was constructed from pGL4.51[*luc*2/CMV/Neo] plasmid vector (Promega, Madison, WI, USA) [56]. MCF-7 cells were transfected with the Luc-reporter vector and liposome (E2431, Promega) using standard protocols. Transfected cells were selected with 1 mg/mL G418 (Sigma-Aldrich, St. Louis, MO, USA) selection antibiotic for two weeks and the resistant colonies were isolated and tested for luciferase activity.

#### 3.5.2. Intracellular Uptake

To evaluate the role of FA in cellular uptake of GOFA, MCF-7 cells were cultured in 0.5 mL α-MEM supplemented with 10% FBS in 24-well culture plates at 1 × 10^4^ cells/well. Cells were grown overnight in a humidified CO_2_ incubator at 37 °C under 5% CO_2_ atmosphere, washed with sterilized PBS and incubated with 0.5 mL QD-labeled GO or GOFA suspension (0.1 mg/mL) for 1 h. Each testing samples were washed with PBS three times and fixed with 4% paraformaldehyde for 15 min, followed by nuclear staining with DAPI. In a separate experiment, the cells were pre-treated with free FA (1 mg/mL) for 1 h to block the folate receptor on cell surface before incubated with QD-labeled GOFA. Possible fluorescence signals from extracellular QD-labeled GOFA bound to the surfaces of MCF-7 cells were quenched by trypan blue dye solution for 15 min. Since trypan blue is excluded from entering live cells, all fluorescence signals observed will be only from GOFA taken intracellularly. The green fluorescence from QD and blue fluorescence from DAPI were examined under a confocal laser scanning microscope (Zeiss LSM 510 Meta, Oberkochen, Germany) with excitation/emission wavelength of 488 nm/500–550 nm and 364 nm/407–482 nm, respectively.

To determine intracellular uptake of GOFA-DOX and release of DOX, MCF-7 cells were cultured in 0.5 mL α-MEM supplemented with 10% FBS in 24-well culture plates at 1 × 10^4^ cells/well. Cells were grown overnight in a humidified CO_2_ incubator at 37 °C under 5% CO_2_ atmosphere, washed with sterilized PBS and incubated with 0.5 mL DOX solution or GOFA-DOX suspension (0.1 mg/mL) for 1 h. Each testing samples were washed with PBS three times and fixed with 4% paraformaldehyde for 15 min, followed by nuclear staining with DAPI (blue) and examination under a confocal laser scanning microscope. The uptake of GOFA-DOX and release of DOX could be visualized by the green fluorescence of QD-labeled GOFA and the red fluorescence of DOX. The excitation wavelength is 543/488/364 nm (red/green/blue) and the emission wavelength is 550–650/500–550/407–482 nm (red/green/blue).

For transmission electron microscope (TEM) analysis, 1 × 10^5^ of MCF-7 cells were grown on ThermoNox (Nunc, Roskilde, Denmark) coverslips and treated with GOFA for 24 h. The cells were fixed in a mixture of 2.5% paraformaldehyde and 2% glutaraldehyde solution for 2 h. Cells were rinsed in 0.1 M sodium cacodylate buffer (pH 7.4) and post-fixed in 1.0% osmium tetroxide for 30 min. The cells were then dehydrated in a graded ethanol series (30%, 50%, 70% with 3% uranyl acetate, 80%, 95% and 100%) for 10 min at each concentration and followed by two changes in 100% propylene oxide. After infiltration and embedding in epoxy resins at 60 °C for 48 h, ultrathin sections (approximately 80 nm) were examined under a FEI/Philips CM 120 TEM (Hillsboro, OR, USA).

#### 3.5.3. In Vitro Cytotoxicity Assessment

For in vitro cytotoxicity tests, MCF-7 cells were cultured in α-MEM supplemented with 10% FBS in a 96-well culture plate at a seeding density of 1 × 10^4^ cells/well and incubated overnight at 37 °C in a humidified 5% CO_2_ atmosphere. After being rinsed with PBS (pH 7.4), the cells were incubated with 200 μL of DOX solutions, GO-DOX or GOFA-DOX suspensions prepared in culture medium containing different DOX concentrations to determine the IC50 (half-maximum inhibitory concentration) value. Cell viability after 24 h was determined using MTT assays by adding 50 μL MTT reagent to each well and incubated for 3 h at 37 °C. After removing the medium and MTT reagent, 200 μL of dimethylsulfoxide was added to the well to dissolve the crystal produced and the absorbance was measured at 540 nm using a Synergy HT microplate reader (BioTek, Winooski, VT, USA). Each experiment was repeated six times. All procedures were finished in conditions devoid of light. Cell viability using cell culture medium and GO (GOFA) were taken as 100% for DOX and GO-DOX (GOFA-DOX), respectively. Control cytotoxicity experiments to confirm the biocompatibility of the drug-free carrier were carried out using MCF-7 cells by following the same procedure as described above within a concentration range of 0.01–100 μg/mL GOFA.

The cytotoxicity of GOFA-DOX/HACPN was determined in a double-chamber dish with MCF-7 cells cultured in α-MEM supplemented with 10% FBS in a 24-well culture plate at a seeding density of 1 × 10^4^ cells/well and incubated overnight at 37 °C in a humidified 5% CO_2_ atmosphere. The GOFA/HACPN solution was prepared by dissolving 0.1 g HACPN in 1 mL GOFA-DOX solution (0.1 mg GOFA in pH 7.4 PBS) with 0, 0.001 or 0.025 mg/mL DOX). 0.2 mL of the GOFA-DOX/HACPN solution was placed in Millicell^®^ cell culture inserts and fitted in the 24-well cell culture plate. The relative cell viability was determined by MTT assays at 24 and 72 h by MTT assays as described above with cell culture medium as control.

#### 3.5.4. Analysis of Apoptosis Using Annexin V and Propidium Iodide Staining

Apoptotic MCF-7 cells were identified with fluorescein isothiocyanate-labeled Annexin V (Annexin V-FITC, BD Biosciences, Franklin Lakes, NJ, USA). Propidium iodide (PI) was also used as a dead cell marker. MCF-7 (5 × 10^5^ cells per well) were seeded in a six-well plate and cultured for 24 h. After treatment with free DOX, GO-DOX and GOFA-DOX for 24 h, the cells were harvested, trypsinized, washed with PBS and incubated with Annexin V-FITC and PI for 15 min at room temperature in the dark. The samples were immediately analyzed with the FACSCalibur flow cytometer (BD Biosciences, Franklin Lakes, NJ, USA) with the CellQuest software.

### 3.6. Animal Studies

#### 3.6.1. Xenograft Tumor Mouse Model

All animal procedures were approved by the Institutional Animal Care and Use Committee of Chang Gung University (IACUC Approval No. CGU14-092). Female nude mice (BALB/cAnN.Cg-Foxn1nu/CrlNarl, 4–6 weeks old, weighed between 20 and 25 g) were purchased from the National Laboratory Animal Center (Taipei, Taiwan) and used for the in vivo animal studies. Mice were cared, housed and maintained in specific sterile environment in the Laboratory Animal Center, Chang Gung University. Estrogen-responsive MCF-7 xenograft tumor model was established and maintained by injecting 5 × 10^6^ MCF-7 cells (in 0.1 mL of Matrigel Matrix High Concentration, BD Biosciences, Franklin Lakes, NJ, USA) subcutaneously into the backs of the 6-week-old mice after anesthetized with 5 mg/kg xylazine (Rompum, Bayer) and 0.8 mg/kg Tiletamin + Zolezepam (Zoletil 50, Virbac) [61]. Animals were used in experiments after 14 days when the tumor volumes reached 60~100 mm^3^.

#### 3.6.2. In Vivo Antitumor Efficacy

Thirty tumor-bearing mice were randomly divided into 5 groups with 6 mice in each group. Group 1, intratumoral injection with 200 μL of saline (control); group 2, intratumoral injection with 200 μL of 10% (*w*/*v*) GOFA/HACPN; group 3, intratumoral injection with 200 μL of DOX solution (30 mg/kg of DOX); group 4, intravenous injection with 200 μL of GOFA-DOX (30 mg/kg of DOX); group 5, intratumoral injection with 200 μL of 10% (*w*/*v*) GOFA-DOX/HACPN (30 mg/kg of DOX). After administration, the tumor size and body weight was monitored continuously for 21 days. Tumor volumes were calculated based on the length and width of tumor (length × (width)^2^/2). The relative of tumor volume (%) was calculated from *V_t_*/*V*_0_, where *V_t_* indicated the tumor volume at time *t* and *V*_0_ indicated the tumor volume at day 0. The relative body weight (%) was calculated according to *W_t_*/*W*_0_, where *W_t_* indicated the weight at time *t* and *W*_0_ indicated the weight at day 0.

#### 3.6.3. Histological, Immunohistochemical, Hematologic and Biochemical Analysis

After sacrificing, tumor tissues were immediately harvested and fixed in 10% phosphate buffered formalin. The tissues were embedded in paraffin and sectioned (8 μm), followed by hematoxylin and eosin (H&E) staining. Paraffin-embedded tumor tissues were stained for proliferating cell nuclear antigen (PCNA) using N-Histofine^®^ MOUSESTAIN KIT (Nichirei Biosciences Inc., Tokyo, Japan) following the manufacturer’s protocol [62]. The primary antibody was mouse monoclonal anti-PCNA antibody (Abcam ab29). 

To evaluate the systemic toxicity of GOFA-DOX/HACPN, major organs including hearts, livers, spleens, lungs and kidneys were harvested before euthanasia and embedded in paraffin and sectioned for H&E staining. Blood samples were collected for hematologic analysis (white blood cell count, red blood cell count, hemoglobin and hematocrit) and biochemical analysis (aspartate aminotransferase, alanine aminotransferase, blood urea nitrogen and creatinine) of major organ functions. The mice in the control group (saline) were used as comparison.

#### 3.6.4. Bioluminescence Imaging (BLI) for In Vivo Evaluation of Anti-Tumor Efficacy

MCF-7/Luc cells were injected subcutaneously into the backs of the 6-week-old mice following and animal were grouped as described for MCF-7 cells. The bioluminescence imaging (BLI) was performed using noninvasive in vivo imaging system (IVIS) (Xenogen IVIS-200, Caliper Life Sciences, Hopkinton, MA, USA). Mice were anesthetized with 1% isoflurane in room air. D-Luciferin (Gold Biotechnology, New Taipei City, Taiwan) in PBS (15 mg/mL) was injected intraperitoneally at a dose of 150 mg/kg and images were acquired to determine the peak bioluminescence. The BLI intensity was determined at baseline (i.e., before treatment) and 21 days after treatment by measuring the total peak bioluminescent signal intensity through standardized regions of interest (ROIs) in tumor by using the Living Image^®^ 4.0 software (PerkinElmer, Waltham, MA, USA). The relative BLI (%) was calculated from the total bioluminescent signal intensity at day 21 normalized by the total signal intensity at baseline.

### 3.7. Statistical Analyses

All data were reported as mean ± standard deviation (SD). Statistical significances were analyzed by Statistical Product and Service Solutions (SPSS) one-way ANOVA Least Significant Difference (LSD) test and differences were considered significant at *p* < 0.05. 

## 4. Conclusions

In conclusion, we have confirmed the synthesis of the nano-sized anticancer drug carrier GOFA, the intracellular uptake of GOFA by endocytosis and the specific targeting effect of GOFA toward MCF-7 breast cancer cells. The high loading capacity of DOX on GOFA in addition to the pH-dependent drug release behavior could facilitate drug release after endocytosis and maintain a high drug concentration cytotoxic to MCF-7 cells. The temperature-sensitive in-situ forming hydrogel HACPN could provide fast sol-gel phase transition kinetics around the physiological temperature. The gelled HACPN could serve as a depot for continuous GOFA-DOX release during hydrogel degradation and offer a facile intratumoral delivery platform for the chemotherapeutic drug. Enhanced cytotoxicity of GOFA-DOX toward MCF-7 was confirmed through MTT assays and flow cytometry analysis, which could be ascribed to the FA-targeting effect. GOFA-DOX/HACPN also showed effective drug dosage and time-dependent cytotoxicity effects in vitro, suggesting its potential for in vivo anticancer therapy. From xenograft tumor mouse model with subcutaneously implanted MCF-7 (MCF-7/Luc) cells, tumor volume measurement and BLI signal intensity revealed the highest anticancer efficiency of GOFA-DOX/HACPN. H&E staining and immunohistochemistry of tumor tissues confirmed this treatment could result in the best effect to induce tumor necrosis and reduction of expression of the tumor cell proliferating marker (PCNA). In addition, no side effects were detected from biopsy of major organs and blood analysis to endorse the safety of this treatment. Taken together, we could conclude the intratumoral delivery of GOFA-DOX/HACPN could be suggested as a safe and effective drug delivery system for breast cancer chemotherapy or potentially also applicable to treatment of other local solid tumors.

## Figures and Tables

**Table 1:** Blood analysis for evaluation of systemic toxicity.

Item	Unit	Control	GOFA-DOX/HACPN
**Hematology**			
WBC	10^3^ cells/μL	5.78 ± 2.34	2.9 ± 0.6 *
RBC	10^6^ cells/μL	8.57 ± 0.51	8.5 ± 0.2 *
HGB	g/dL	13.30 ± 0.87	13.2 ± 0.5 *
HCT	%	41.77 ± 2.23	39.1 ± 2.5 *
PLT	10^3^ cells/μL	334.30 ± 29.8	279.5 ± 37.5 *
**Clinical Chemistry**			
AST	U/L	254.5 ± 19.1	294 ± 39.4 *
ALT	U/L	102.50 ± 0.71	113.8 ± 51.3 *
BUN	mg/dL	28.40 ± 3.54	32.1 ± 3.1 *
CREA	mg/dL	0.12 ± 0.01	0.13 ± 0.01 *

WBC: white blood cell; RBC: red blood cell; HGB: hemoglobin; HCT: hematocrit; PLT: platelet; AST: aspartate transaminase; ALT: alanine transaminase; BUN: blood urea nitrogen; CREA: creatinine Values are means ± standard deviation (SD) of six independent measurements. * *p* > 0.05 compared with control.
